# Additive-multiplicative hazards regression models for interval-censored semi-competing risks data with missing intermediate events

**DOI:** 10.1186/s12874-019-0678-z

**Published:** 2019-03-06

**Authors:** Jinheum Kim, Jayoun Kim, Seong W. Kim

**Affiliations:** 1 Department of Applied Statistics, University of Suwon, Suwon, 18323 South Korea; 2 Medical Research Collaborating Center, Seoul National University Hospital, Seoul, 03080 South Korea; 3 Department of Applied Mathematics, Hanyang University, Ansan, 15588 South Korea

**Keywords:** Additive and multiplicative hazards model, Interval censoring, log-normal frailty, Missing intermediate event, Multi-state model, Semi-competing risks data

## Abstract

**Background:**

In clinical trials and survival analysis, participants may be excluded from the study due to withdrawal, which is often referred to as lost-to-follow-up (LTF). It is natural to argue that a disease would be censored due to death; however, when an LTF is present it is not guaranteed that the disease has been censored. This makes it important to consider both cases; the disease is censored or not censored. We also note that the illness process can be censored by LTF. We will consider a multi-state model in which LTF is not regarded as censoring but as a non-fatal event.

**Methods:**

We propose a multi-state model for analyzing semi-competing risks data with interval-censored or missing intermediate events. More precisely, we employ the additive and multiplicative hazards model with log-normal frailty and construct the conditional likelihood to estimate the transition intensities among states in the multi-state model. Marginalization of the full likelihood is accomplished using adaptive importance sampling, and the optimal solution of the regression parameters is achieved through the iterative quasi-Newton algorithm.

**Results:**

Simulation is performed to investigate the finite-sample performance of the proposed estimation method in terms of the relative bias and coverage probability of the regression parameters. The proposed estimators turned out to be robust to misspecifications of the frailty distribution. PAQUID data have been analyzed and yielded somewhat prominent results.

**Conclusions:**

We propose a multi-state model for semi-competing risks data for which there exists information on fatal events, but information on non-fatal events may not be available due to lost to follow-up. Simulation results show that the coverage probabilities of the regression parameters are close to a nominal level of 0.95 in most cases. Regarding the analysis of real data, the risk of transition from a healthy state to dementia is higher for women; however, the risk of death after being diagnosed with dementia is higher for men.

## Background

In classical time-to-event or survival analysis, subjects are under risk for one fatal event. However, subjects do not fail from just one certain type of event in some applications, but are under risk of failing from two or more mutually exclusive types of events. When an individual is under risk of failing from two different types of event, these different event types are called competing risks. One of the events censors the other, and vice versa, in these competing risks frameworks [1–3]. However, many clinical trials have revealed that a subject can experience both a non-fatal event (e.g., a disease or relapse) and a fatal event (e.g., death), where the fatal event censors the non-fatal event but not vice versa. We call these types of data semi-competing risks data [4–6].

In clinical trials, the occurrence of a non-fatal event can be detected in conjunction with possibly incessant monitoring during periodic follow-up. For illustration purposes of our methodologies, a dataset named PAQUID (Personnes Agées Quid) is analyzed to investigate the meaningful prognostic factors associated with dementia. These data were initially analyzed by [7] using the conventional Cox model [8, 9]. Complete descriptions of the PAQUID data can be found in Conclusions section. In this paper, we employ a semi-competing risks model where death may occur after dementia has occurred (i.e., been diagnosed), but death censors the disease. An illness-death model [10] is perhaps one of the most commonly and frequently used semi-competing risks models. Many studies have been conducted under semi-competing risks frameworks [4, 5, 11].

As shown in the PAQUID data, dementia can be censored informatively by death. Furthermore, an additional informative censoring process can also occur. That is, participants may be excluded from the study due to withdrawal, which is often referred to as lost-to-follow-up (LTF). It is clear that dementia would be censored due to death; however, when an LTF is present it is not guaranteed that dementia has been censored. This makes it important to consider both cases; dementia is censored or not censored. We also note that the illness process (dementia) can be censored by LTF. This forces us to consider a multi-state model in which LTF is not regarded as censoring but as a non-fatal event. Considerable studies have utilized this multi-state model. For example, [12] proposed a nonparametric method to estimate the survival function associated with disease occurrences, while [6, 13] used the Cox proportional hazards model [8, 9] to estimate regression coefficients.

In the meantime, most non-fatal events are observed periodically. That is, the event time is not observed exactly but lies on an interval of the form (*L*,*R*], where *L* is the last time a subject visited without possessing a disease and *R* is the first time that the subject was diagnosed with a disease. This type of censoring is called interval censoring. We could emulate what [6] did and assume that a non-fatal event of a subject occurs uniformly on the interval (*L*,*R*]. However, using the methods proposed by [14, 15], we instead partition the interval (*L*,*R*] into a few sub-intervals, in which a non-fatal event can occur. Ultimately, different weights can be assigned to each sub-interval. Thus, the former method corresponds to an unconditional probability approach with equal weight on all of the sub-intervals, whereas the latter utilizes a conditional probability approach with a specific weight, depending on the sub-interval.

In our study, we use the latter method to deal with non-fatal events that are interval-censored on an interval. In addition, we propose an additive-multiplicative model by combining the Cox proportional hazards model with the additive risk model of [16], in accordance with a multi-state model. The additive-multiplicative model was initially introduced by [17] and has since been developed by a number of researchers. Scheike and Zhang [19] incorporated time-varying covariates for the additive part and time-independent covariates for the multiplicative part. On the other hand, [18] estimated relevant parameters by considering time-varying covariates for both additive and multiplicative parts. We also consider the frailty effect as a latent variable to incorporate possible connections between events; this is done because each individual is exposed to several events, including the occurrence of illness, LTF, and death.

The rest of the paper is organized as follows. First, we explain notations and procedures for parameter estimation along with the proposed models. Second, extensive simulation studies are presented to investigate the model performances in terms of the relative bias and coverage probability of the proposed estimates. We also provide the results of real data analysis. Finally, we present a summary and concluding remarks, including some of the drawbacks of the proposed models and directions for future research.

## Methods

### Models

**Fig. 1:**
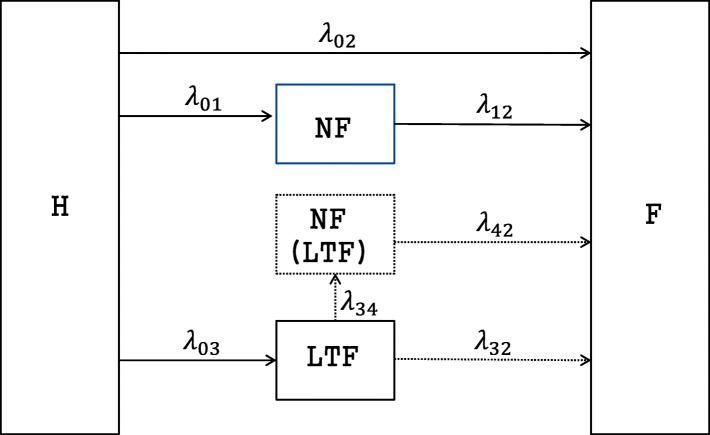
Five-state model

As depicted in Fig. 1, the proposed model in this study consists of five states: healthy (H), non-fatal (NF), fatal (F), lost-to-follow-up (LTF), and unobserved non-fatal (NF(LTF)). Each state is denoted by numbers 0 through 4, respectively. A total of seven possible transitions exists in the model: 0→1, 0→2, 0→3, 1→2, 3→2, 3→4, and 4→2. However, among these transitions, both 3→4 and 4→2 (displayed as dotted lines in Fig. 1 are unobservable and should be regarded as potential transitions.

Let *t* be the time from study entry. Additionally, *S*_*t*_ is defined as the state that each subject can take at *t*≥0. Then, *S*_*t*_∈{0,1,2,3,4}. Let $\mathcal {A}=\{(r,s):(r,s)=(0,1), (0,2), (0,3), (1,2), (3,2), (3,4), (4,2)\}$. Also, define *λ*_*rs*_(*t*) to be the transition intensity from states *r* to *s* at time *t*. That is,

$$\begin{array}{@{}rcl@{}} \lambda_{rs}(t)={\lim}_{dt\rightarrow0}\frac{\Pr(S_{t+dt}=s|S_{t}=r)}{dt}\ \ \text{for }\ \ (r,s)\in\mathcal{A}, \end{array} $$

and *λ*_*rs*_(*t*)=0 for $(r,s)\notin \mathcal {A}$. As mentioned above, the data corresponding to transitions 3→4 and 4→2 are not observable, requiring the following assumptions for *λ*_34_(*t*) and *λ*_42_(*t*):

1 $$\begin{array}{*{20}l} \lambda_{34}(t) &=& \lambda_{01}(t), \ t\ge0,  \end{array} $$

2 $$\begin{array}{*{20}l} \lambda_{42}(t) &=& \lambda_{12}(t),\ t\ge0.  \end{array} $$

Assumptions (1) and (2) imply that the transition intensities of H to NF and NF to F may be the same, irrespective of the occurrence of LTF. As mentioned in Background section, given covariates ***z***=(*z*_1_,*z*_2_,…,*z*_*p*_)^′^ and ***w***=(*w*_1_,*w*_2_,…,*w*_*d*_)^′^, along with frailty *u*, we consider additive and multiplicative models defined as

3 $$ {\begin{aligned} \lambda_{rs}(t|{\boldsymbol{z}},{\boldsymbol{w}},u)=\eta \left({\boldsymbol{\beta}}_{rs}^{\prime}{\boldsymbol{z}} + \exp \left({\boldsymbol{\alpha}}_{rs}^{\prime}{\boldsymbol{w}}\right)\theta_{rs}\gamma_{rs}t^{\gamma_{rs} - 1}\right)\ \ \text{for }\ \ (r,s)\in\mathcal{A}, \end{aligned}}  $$

where *θ*_*rs*_(>0) and *γ*_*rs*_(>0) are the scale and shape parameters of a Weibull distribution, respectively, and ***β***_*rs*_ and ***α***_*rs*_ are vectors of the regression coefficients for the additive and multiplicative parts, respectively. Moreover, *η*= exp(*u*) is the frailty for a log-normal distribution and *u* is assumed to follow a normal distribution with a mean of zero and variance *σ*^2^. Thus, we use a Weibull distribution as a baseline transition intensity and impose a multiplicative frailty effect on the transition intensities. Since the parameters *θ*_34_,*θ*_42_,*γ*_34_,*γ*_42_,***α***_34_,***α***_42_,***β***_34_, and ***β***_42_ related to transitions 3→4 and 4→2 should satisfy the assumptions in (1) and (2), the parameter vector estimated for the model in (3) is ***ζ***=(***θ***^∗^,***γ***^∗^,***α***^∗^,***β***^∗^,*σ*^2^)^′^, where ***θ***^∗^=(*θ*_01_,*θ*_02_,*θ*_03_,*θ*_12_,*θ*_32_), ***γ***^∗^=(*γ*_01_,*γ*_02_,*γ*_03_,*γ*_12_,*γ*_32_), ${\boldsymbol {\alpha }}^{*} =\left ({\boldsymbol {\alpha }}_{01}^{\prime }, {\boldsymbol {\alpha }}_{02}^{\prime }, {\boldsymbol {\alpha }}_{03}^{\prime }, {\boldsymbol {\alpha }}_{12}^{\prime }, {\boldsymbol {\alpha }}_{32}^{\prime }\right)$, and ${\boldsymbol {\beta }}^{*} = \left ({\boldsymbol {\beta }}_{01}^{\prime }, {\boldsymbol {\beta }}_{02}^{\prime }, {\boldsymbol {\beta }}_{03}^{\prime }, {\boldsymbol {\beta }}_{12}^{\prime }, {\boldsymbol {\beta }}_{32}^{\prime }\right)$. According to the model in (3), the cumulative hazard functions *H*_*k*_(*t*_1_,*t*_2_) (*k*=0,1,3,4) for leaving state *k* between *t*_1_ and *t*_2_ can be represented as

$$\begin{array}{@{}rcl@{}} H_{0}(t_{1},t_{2}|{\boldsymbol{z}},{\boldsymbol{w}},u) &=& \int_{t_{1}}^{t_{2}}\{ \lambda_{01}(s|{\boldsymbol{z}},{\boldsymbol{w}},u) + \lambda_{02}(s|{\boldsymbol{z}},{\boldsymbol{w}},u) + \lambda_{03}(s|{\boldsymbol{z}},{\boldsymbol{w}},u)\} ds\\ &=& \sum_{r=1}^{3} \eta\left\{ \left({\boldsymbol{\beta}}_{0r}^{\prime}{\boldsymbol{z}}\right)(t_{2} - t_{1}) + \exp \left({\boldsymbol{\alpha}}_{0r}^{\prime}{\boldsymbol{w}}\right)\theta_{0r}\left(t_{2}^{\gamma_{0r}} - t_{1}^{\gamma_{0r}}\right)\right\}\!,\\ H_{1}(t_{1},t_{2}|{\boldsymbol{z}},{\boldsymbol{w}},u) &=& \int_{t_{1}}^{t_{2}} \lambda_{12}(s|{\boldsymbol{z}},{\boldsymbol{w}},u) ds\\ &=& \eta\left\{ \left({\boldsymbol{\beta}}_{12}^{\prime}{\boldsymbol{z}}\right)(t_{2} - t_{1}) + \exp \left({\boldsymbol{\alpha}}_{12}^{\prime}{\boldsymbol{w}}\right)\theta_{12}\left(t_{2}^{\gamma_{12}} - t_{1}^{\gamma_{12}}\right)\right\}, \end{array} $$

$$ \begin{aligned} H_{3}(t_{1},t_{2}|{\boldsymbol{z}},{\boldsymbol{w}},u) &= \int_{t_{1}}^{t_{2}}\{\lambda_{32}(s|{\boldsymbol{z}},{\boldsymbol{w}},u) + \lambda_{34}(s|{\boldsymbol{z}},{\boldsymbol{w}},u) \}ds \\ &= \eta\big\{ \left({\boldsymbol{\beta}}_{32}^{\prime}{\boldsymbol{z}}\right)(t_{2} - t_{1}) + \exp \left({\boldsymbol{\alpha}}_{32}^{\prime}{\boldsymbol{w}}\right)\theta_{32}\left(t_{2}^{\gamma_{32}} - t_{1}^{\gamma_{32}}\right) \\ &\quad+ \left({\boldsymbol{\beta}}_{34}^{\prime}{\boldsymbol{z}}\right)(t_{2} - t_{1}) + \exp \left({\boldsymbol{\alpha}}_{34}^{\prime}{\boldsymbol{w}}\right)\theta_{34}\left(t_{2}^{\gamma_{34}} - t_{1}^{\gamma_{34}}\right) \big\},\\ H_{4}(t_{1},t_{2}|{\boldsymbol{z}},{\boldsymbol{w}},u) &= \int_{t_{1}}^{t_{2}} \lambda_{42}(s|{\boldsymbol{z}},{\boldsymbol{w}},u) ds\\ &= \eta\left\{ \left({\boldsymbol{\beta}}_{42}^{\prime}{\boldsymbol{z}}\right)(t_{2} - t_{1}) + \exp \left({\boldsymbol{\alpha}}_{42}^{\prime}{\boldsymbol{w}}\right)\theta_{42}\left(t_{2}^{\gamma_{42}} - t_{1}^{\gamma_{42}}\right)\right\}. \end{aligned} $$

Based on Assumptions (1) and (2), we note that ***β***_34_=***β***_01_, ***β***_42_=***β***_12_, ***α***_34_=***α***_01_, ***α***_42_=***α***_12_, *θ*_34_=*θ*_01_, *θ*_42_=*θ*_12_, *γ*_34_=*γ*_01_, and *γ*_42_=*γ*_12_ in the equations of *H*_3_ and *H*_4_.

### Parameter estimation

As shown in Fig. 1, a total of six routes can be experienced by a subject from the beginning to the end of the study. These are route 1 (0 → 0), route 2 (0 → 2), route 3 (0 → 1), route 4 (0 → 1 → 2), route 5 (0 → 3), and route 6 (0 → 3 → 2). In particular, route 5 can be classified into two paths, i.e., 0 → 3 and 0 → 3 → 4, depending on whether or not a subject experiences the unobservable NF state. Similarly, route 6 can be classified into two paths: 0 → 3 → 2 and 0 → 3 → 4 → 2. We introduce notations to define the likelihood associated with each route. Consider three random variables *R*, *L*, and *T*, each of which represents a time from the start of the study until the occurrence of a non-fatal event, LTF, and a fatal event, respectively. Furthermore, let $\mathcal {H}_{0}(s)$ be the set of subjects staying in state 0 at time *s*. That is,

$$\begin{array}{@{}rcl@{}} \mathcal{H}_{0}(s)=\{R\wedge L\wedge T>s\}. \end{array} $$

Let $\mathcal {H}_{3,f}(s)$ be the set of subjects who have already experienced LTF at time *f* and remain in state 3 at time *s*. Then,

$$\begin{array}{@{}rcl@{}} \mathcal{H}_{3,f}(s)=\{L=f,R\wedge T>s, f\le s\}. \end{array} $$

Now, let *e*_*i*_ be the entry time of study, *a*_*i*_ be the last time the *i*^th^ subject visited before a non-fatal event was observed, and *b*_*i*_ be the first time a non-fatal event is observed by the *i*^th^ subject for *i*=1,2,…,*n*. Consider an indicator function *I*_*ij*_, which is 1 if subject *i* follows route *j* and zero otherwise for *j*=1,2,…,6. Let $\mathcal {B}_{j}=\{i:I_{ij}=1\}$. For subject $i\in \mathcal {B}_{1}\cup \mathcal {B}_{2}$, we have *a*_*i*_,*b*_*i*_≥*t*_*i*_; this is the case because a non-fatal event has not been observed before time *t*_*i*_. For subject $i\in \mathcal {B}_{3}\cup \mathcal {B}_{4}$, we have *a*_*i*_<*b*_*i*_≤*t*_*i*_; this is the case because a non-fatal event has occurred between *a*_*i*_ and *b*_*i*_. When subject *i* is a member of $\mathcal {B}_{5}\cup \mathcal {B}_{6}$, LTF has occurred at time *a*_*i*_, which yields *a*_*i*_<*t*_*i*_; however, *b*_*i*_<*t*_*i*_ or *b*_*i*_≥*t*_*i*_, depending on whether an unobservable non-fatal event has occurred. Thus, *t*_*i*_ would be a censoring time for $i\in \mathcal {B}_{1}\cup \mathcal {B}_{3}\cup \mathcal {B}_{5}$, whereas it would be a time of death for $i\in \mathcal {B}_{2}\cup \mathcal {B}_{4}\cup \mathcal {B}_{6}$. Therefore, likelihood functions *Q*_1_ and *Q*_2_ can be constructed for routes 1 and 2, respectively. These are given as follows:

4 $$\begin{array}{@{}rcl@{}} Q_{i1} &=& \Pr\left(R_{i}\wedge L_{i}\wedge T_{i}>t_{i}|\mathcal{H}_{0}(e_{i}),{\boldsymbol{z}}_{i}, {\boldsymbol{w}}_{i}, u_{i}\right) \\ &=& \exp\{-H_{0}(e_{i},t_{i}|{\boldsymbol{z}}_{i}, {\boldsymbol{w}}_{i}, u_{i})\}, \ i\in\mathcal{B}_{1}. \end{array} $$

5 $$\begin{array}{@{}rcl@{}} Q_{i2} &=& \Pr(T=t_{i}, R\wedge L>t_{i}|\mathcal{H}_{0}(e_{i}),{\boldsymbol{z}}_{i}, {\boldsymbol{w}}_{i}, u_{i}) \\ &=& Q_{i1}\times\lambda_{02}(t_{i}|{\boldsymbol{z}}_{i}, {\boldsymbol{w}}_{i}, u_{i}),\ i\in\mathcal{B}_{2}. \end{array} $$

Likelihood functions can also be constructed for routes 3 and 4:

6 $$\begin{array}{@{}rcl@{}} Q_{i3}^{*} &=& \Pr(R_{i}\in(a_{i},b_{i}],L_{i}>t_{i},T_{i}>t_{i}|\mathcal{H}_{0}(e_{i}), {\boldsymbol{z}}_{i}, {\boldsymbol{w}}_{i}, u_{i}) \\ &=&\int_{a_{i}}^{b_{i}}\bigg[\exp\{-H_{0}(e_{i},s|{\boldsymbol{z}}_{i}, {\boldsymbol{w}}_{i}, u_{i})\}\lambda_{01}(s|{\boldsymbol{z}}_{i}, {\boldsymbol{w}}_{i}, u_{i})\\ &&\quad\times\exp\{-H_{1}(s,t_{i}|{\boldsymbol{z}}_{i}, {\boldsymbol{w}}_{i}, u_{i})\}\bigg]ds. \end{array} $$

7 $$\begin{array}{@{}rcl@{}} Q_{i4}^{*}&\!=& \Pr(R_{i}\!\in\!(a_{i},b_{i}],L_{i}\!>\!t_{i},R_{i}\!<T_{i}=t_{i}|\mathcal{H}_{0}(e_{i}), {\boldsymbol{z}}_{i}, {\boldsymbol{w}}_{i}, u_{i}) \\ &\!=& Q_{i3}^{*}\times\lambda_{12}(t_{i}|{\boldsymbol{z}}_{i}, {\boldsymbol{w}}_{i}, u_{i}),\ i\in\mathcal{B}_{4}.  \end{array} $$

Equations (6) and (7) are derived by assuming that a non-fatal event of subject *i* in the set $\mathcal {B}_{3}\cup \mathcal {B}_{4}$ can occur uniformly on the interval (*a*_*i*_,*b*_*i*_] [6]. However, we partition the interval (*a*_*i*_,*b*_*i*_] into several sub-intervals where non-fatal events could occur and assign different weights to each interval [15].

- Let $R_{i^{\prime }}\in (a_{i^{\prime }},b_{i^{\prime }}]$ be an interval for the occurrence of non-fatal events associated with subjects in routes 3 or 4. Let *s*_1_ be the smallest value among all $b_{i^{\prime }}$’s for subjects in the set $\mathcal {B}_{3}\cup \mathcal {B}_{4}$. Let *s*_2_ be the smallest value among all $b_{i^{\prime }}$’s corresponding to subjects having $a_{i^{\prime }}$ greater than or equal to *s*_1_. This process is repeated until we have no subjects with $a_{i^{\prime }}$ greater than or equal to *s*_*m*_ (*m*=1,2,…). Thus, we can have a refined set of time points
  $$\begin{array}{@{}rcl@{}} 0=s_{0}< s_{1}< s_{2}<\cdots< s_{l}< s_{l+1}=\infty. \end{array} $$
- We can define the weight $w_{i^{\prime }m}$ at time *s*_*m*_ (*m*=1,2,…) for subject *i*^′^ in the set $\mathcal {B}_{3}\cup \mathcal {B}_{4}:$
  8 $$ {\begin{aligned} w_{i^{\prime}m} = \frac{d_{i^{\prime}m}\exp\left\{-H_{0}\left(e_{i^{\prime}},s_{m}|{\boldsymbol{w}}_{i^{\prime}}, {\boldsymbol{z}}_{i^{\prime}}, u_{i^{\prime}}\right)\right\}\lambda_{01}(s_{m}|{\boldsymbol{w}}_{i^{\prime}}, {\boldsymbol{z}}_{i^{\prime}}, u_{i^{\prime}})}{\sum_{m^{\prime}=1}^{l} d_{i^{\prime}m^{\prime}}\exp\left\{-H_{0}(e_{i^{\prime}},s_{m^{\prime}}|{\boldsymbol{w}}_{i^{\prime}}, {\boldsymbol{z}}_{i^{\prime}}, u_{i^{\prime}})\right\}\lambda_{01}(s_{m^{\prime}}|{\boldsymbol{w}}_{i^{\prime}}, {\boldsymbol{z}}_{i^{\prime}}, u_{i^{\prime}})}, \end{aligned}}  $$

where $d_{i^{\prime }m} = I(s_{m}\in (a_{i^{\prime }},b_{i^{\prime }}])$. Subsequently, likelihood functions incorporated with weight $w_{i^{\prime }m}$ in (8) for routes 3 and 4 are given by

9 $$ {\begin{aligned} Q_{i3}=& \sum_{m=1}^{l}d_{im}w_{im}\exp\{-H_{0}(e_{i},s_{m}|{\boldsymbol{z}}_{i}, {\boldsymbol{w}}_{i}, u_{i})\}\lambda_{01}(s_{m}|{\boldsymbol{z}}_{i}, {\boldsymbol{w}}_{i}, u_{i})\\ & \times \exp\{-H_{1}(s_{m}, t_{i}|{\boldsymbol{z}}_{i}, {\boldsymbol{w}}_{i}, u_{i})\},\ i\in\mathcal{B}_{3}.  \end{aligned}}  $$

10 $$\begin{array}{@{}rcl@{}} Q_{i4} = Q_{i3}\times\lambda_{12}(t_{i}|{\boldsymbol{z}}_{i}, {\boldsymbol{w}}_{i}, u_{i}),\ i\in\mathcal{B}_{4}.  \end{array} $$

Finally, likelihood functions for routes 5 and 6 are given by

11 $$\begin{array}{@{}rcl@{}} Q_{i5}&=& \Pr(R_{i}\wedge T_{i}>t_{i}|\mathcal{H}_{3,a_{i}}(a_{i}),{\boldsymbol{z}}_{i}, {\boldsymbol{w}}_{i}, u_{i}) \\ &&+ \Pr(R_{i}\in(a_{i},t_{i}], T_{i}>t_{i}|\mathcal{H}_{3,a_{i}}(a_{i}),{\boldsymbol{z}}_{i}, {\boldsymbol{w}}_{i}, u_{i}) \\ &=& \exp\{-H_{0}(e_{i},a_{i}|{\boldsymbol{z}}_{i}, {\boldsymbol{w}}_{i}, u_{i})\}\lambda_{03}(a_{i}|{\boldsymbol{z}}_{i}, {\boldsymbol{w}}_{i}, u_{i})\\ &&\times \bigg[ \exp\{-H_{3}(a_{i},t_{i}|{\boldsymbol{z}}_{i}, {\boldsymbol{w}}_{i}, u_{i})\} \\ &&+ \int_{a_{i}}^{t_{i}}\exp\{-H_{3}(a_{i},s|{\boldsymbol{z}}_{i}, {\boldsymbol{w}}_{i}, u_{i})\}\lambda_{34}(s|{\boldsymbol{z}}_{i}, {\boldsymbol{w}}_{i}, u_{i}) \\ &&\times \exp\{-H_{4}(s,t_{i}|{\boldsymbol{z}}_{i}, {\boldsymbol{w}}_{i}, u_{i})\} ds \bigg], \ i\in\mathcal{B}_{5}, \end{array} $$

12 $$ {\begin{aligned} Q_{i6}=&\Pr(R_{i}>T_{i},\ T_{i}=t_{i}|\ \mathcal{H}_{3,\ a_{i}}(a_{i}),{\boldsymbol{z}}_{i}, {\boldsymbol{w}}_{i}, u_{i}) \\ &+ \Pr(R_{i} \in(a_{i},\ t_{i}],\ R_{i}<T_{i}=t_{i}|\mathcal{H}_{3,a_{i}}(a_{i}),{\boldsymbol{z}}_{i}, {\boldsymbol{w}}_{i}, u_{i}) \\ =& \exp\{-H_{0}(e_{i},a_{i}|{\boldsymbol{z}}_{i}, {\boldsymbol{w}}_{i}, u_{i})\}\lambda_{03}(a_{i}|{\boldsymbol{z}}_{i}, {\boldsymbol{w}}_{i}, u_{i})\\ &\times\bigg [ \exp\{-H_{3}(a_{i},t_{i}|{\boldsymbol{z}}_{i}, {\boldsymbol{w}}_{i}, u_{i})\} \lambda_{32}(t_{i}|{\boldsymbol{z}}_{i}, {\boldsymbol{w}}_{i}, u_{i})\\ &+\bigg\{\int_{a_{i}}^{t_{i}}\exp\{-H_{3}(a_{i},s|{\boldsymbol{z}}_{i}, {\boldsymbol{w}}_{i}, u_{i})\}\lambda_{34}(s|{\boldsymbol{z}}_{i}, {\boldsymbol{w}}_{i}, u_{i}) \\ &\times \exp\{-H_{4}(s,t_{i}|{\boldsymbol{z}}_{i}, {\boldsymbol{w}}_{i}, u_{i}) \} ds \bigg\} \lambda_{42}(t_{i}|{\boldsymbol{z}}_{i}, {\boldsymbol{w}}_{i}, u_{i}) \bigg],\ i\in\mathcal{B}_{6}.  \end{aligned}}  $$

Therefore, based on Eqs. (4)-(5) and (9)-(12), the likelihood function for the parameter vector ***ζ*** is

13 $$\begin{array}{@{}rcl@{}} L({\boldsymbol{\zeta}})=\prod_{i=1}^{n}\left\{\prod_{j=1}^{6}Q_{ij}^{I_{ij}}\right\} \phi\left(0,\sigma^{2};u_{i}\right), \end{array} $$

where *ϕ*(·) is the probability density function of a normal distribution with a mean of zero and variance *σ*^2^. In our analysis, we use the NLMIXED procedure of the SAS software to estimate ***ζ***. For the sake of parameter estimation procedures, we define the marginal likelihood as

$$\begin{array}{@{}rcl@{}} m({\boldsymbol{\zeta}})=\int\cdots\int L({\boldsymbol{\zeta}}){du}_{1}\cdots {du}_{n}. \end{array} $$

Then, we find the value of ***ζ*** that minimizes *f*(***ζ***)=− log*m*(***ζ***), which is referred to as $\hat {{\boldsymbol {\zeta }}}$. Consequently, the inverse of the Hessian matrix evaluated at $\hat {{\boldsymbol {\zeta }}}$ is defined as the estimated variance-covariance matrix of $\hat {{\boldsymbol {\zeta }}}$. Numerical integration is required for the frailty distribution. For this purpose, we use the adaptive importance sampling [20]. Finally, we employ quasi-Newton optimization, which utilizes the gradient vector and the Hessian matrix of *f*(***ζ***), to achieve the optimal solution of ***ζ***.

## Results

### Simulation studies

Extensive simulation is performed to investigate the finite-sample properties of the estimators proposed in Methods section. As mentioned earlier, we assume a Weibull distribution with a shape parameter of *γ*_*rs*_=1 as the baseline transition intensity and a log-normal distribution for frailty *η*= exp(*u*), where *u* is generated from a normal distribution with a mean of zero and a variance of 0.01. Furthermore, we use a binary covariate for *z* (generated from a Bernoulli trial with a success probability of 0.5) and a continuous covariate for *w* (generated from a standard normal distribution). We fix the sample size *n* at 200 and the censoring time *C* at 365. A total of 500 replications is used in our simulations. The following presents the details related to the generation of random variates for the *i*^th^ (*i*=1,2,…,*n*) subject.

- Step 0: We may allow the total number of occurrences for non-fatal events to be 24 times in a 12-month period, such as 15,31,…,349,365 days. However, the actual visiting time of each subject can be different from the designated times. Hence, we add random numbers, generated from a normal distribution with a mean of zero and a variance of 9, to each designated time point. Subsequently, the actual observed time points will be defined as
  $$\begin{array}{@{}rcl@{}} 0= l_{0} < l_{1i} < \cdots < l_{23,i} < l_{24}=366. \end{array} $$
  Let *u*_01*i*_, *u*_02*i*_, and *u*_03*i*_ be random numbers generated from a uniform distribution on the interval (0,1). Additionally, let *R*_*i*_, *T*_*i*_, and *L*_*i*_ be, respectively, the roots *s* of the equations:
  $$\begin{array}{*{20}l} \Lambda_{01}(s|z_{i}, w_{i},u_{i}) + \log(1-u_{01i})&=0,\\ \Lambda_{02}(s|z_{i}, w_{i},u_{i}) + \log(1-u_{02i})&=0,\\ \text{and} \Lambda_{03}(s|z_{i}, w_{i},u_{i}) + \log(1-u_{03i})&=0, \end{array} $$
  where
  $$\begin{array}{@{}rcl@{}} \Lambda_{0j}(s|z_{i}, w_{i},u_{i}) = \eta_{i}\left[\left(\beta_{0j} z_{i}\right) s + \exp\{\alpha_{0j} w_{i}\} \theta_{0j} s^{\gamma_{0j}}\right]\ \text{for}\ j=1,2,3. \end{array} $$
- Step 1: If *C*≤*R*_*i*_∧*T*_*i*_∧*L*_*i*_, then the *i*^th^ subject is defined as being censored without experiencing a non-fatal event, i.e., $i \in \mathcal {B}_{1}$. If *T*_*i*_=*R*_*i*_∧*T*_*i*_∧*L*_*i*_, then the *i*^th^ subject is defined as being dead without experiencing a non-fatal event, i.e., $i \in \mathcal {B}_{2}$. However, if *R*_*i*_=*R*_*i*_∧*T*_*i*_∧*L*_*i*_, proceed to Step 2, and if *L*_*i*_=*R*_*i*_∧*T*_*i*_∧*L*_*i*_, proceed to Step 3.
- Step 2: Let *u*_12*i*_ be a random number generated from a uniform distribution on the interval (1− exp{*Λ*_12_(*R*_*i*_|*z*_*i*_,*w*_*i*_,*u*_*i*_)},1), where
  $$\begin{array}{@{}rcl@{}} \Lambda_{12}(s|z_{i},w_{i},u_{i})=\eta_{i}\left[(\beta_{12} z_{i}) s + \exp\{\alpha_{12}w_{i}\} \theta_{12}s^{\gamma_{12}}\right]. \end{array} $$
  Redefine *T*_*i*_ as the root *s* of the equation,
  $$\begin{array}{@{}rcl@{}} \Lambda_{12}(s|z_{i},w_{i},u_{i}) + \log(1-u_{12i})=0. \end{array} $$
  If *C*≤*T*_*i*_, then the *i*^th^ subject is defined as being censored after experiencing a non-fatal event, i.e., $i \in \mathcal {B}_{3}$. Otherwise, the *i*^th^ subject is defined as being dead at time *T*_*i*_ after experiencing a non-fatal event, i.e., $i \in \mathcal {B}_{4}$. Moreover,
  - If *R*_*i*_∈(0,*l*_1*i*_), let *a*_*i*_=0 and *b*_*i*_=*l*_1*i*_. If *R*_*i*_∈(*l*_*k*−1,*i*_,*l*_*ki*_), let *a*_*i*_=*l*_*k*−1,*i*_ and *b*_*i*_=*l*_*ki*_ for *k*=2,3,…,23.
  - However, if *R*_*i*_∈(*l*_23,*i*_,*C*), the type of path should be redefined because a non-fatal event for the subject did not occur before the time of the last observation. Thus, if *C*≤*T*_*i*_, the *i*^th^ subject is defined as being censored without experiencing a non-fatal event, i.e., $i \in \mathcal {B}_{1}$. Otherwise, the *i*^th^ subject is defined as being dead at time *T*_*i*_ without experiencing a non-fatal event, i.e., $i \in \mathcal {B}_{2}$.
- Step 3: Let *u*_32*i*_ and *u*_34*i*_ be random numbers generated from uniform distributions on the intervals (1− exp{*Λ*_32_(*L*_*i*_|*z*_*i*_,*w*_*i*_,*u*_*i*_)},1) and (1− exp{*Λ*_34_(*L*_*i*_|*z*_*i*_,*w*_*i*_,*u*_*i*_)},1), respectively, where
  $$\begin{array}{@{}rcl@{}} \Lambda_{3j}(s|z_{i},w_{i},u_{i})=\eta_{i}\left[(\beta_{3j} z_{i}) s + \exp\{\alpha_{3j}w_{i}\} \theta_{3j}s^{\gamma_{3j}}\right],\ \text{for}\ j=2,4. \end{array} $$
  Now redefine *R*_*i*_ and *T*_*i*_ as the roots *s* of the equations:
  $$\begin{array}{*{20}l} \Lambda_{32}(s|z_{i},w_{i},u_{i}) + \log(1-u_{32i})&=0 \\ \text{and} \Lambda_{34}(s|z_{i},w_{i},u_{i}) + \log(1-u_{34i})&=0, \end{array} $$
  respectively. If *C*≤*R*_*i*_∧*T*_*i*_, the *i*^th^ subject is defined as being censored without experiencing a non-fatal event after LTF, i.e., $i \in \mathcal {B}_{5}$. If *T*_*i*_≤*R*_*i*_, then the *i*^th^ subject is defined as being dead without experiencing a non-fatal event after LTF, i.e., $i \in \mathcal {B}_{6}$. However, if *R*_*i*_<*T*_*i*_, move to Step 4.
- Step 4: Let *u*_42*i*_ be a random number generated from a uniform distribution on the interval (1− exp{*Λ*_42_(*R*_*i*_|*z*_*i*_,*w*_*i*_,*u*_*i*_)},1), where
  $$\begin{array}{@{}rcl@{}} \Lambda_{42}(s|z_{i},w_{i},u_{i})=\eta_{i}\left[(\beta_{42} z_{i}) s + \exp\{\alpha_{42}w_{i}\} \theta_{42}s^{\gamma_{42}}\right]. \end{array} $$
  Redefine *T*_*i*_ as the root *s* of the equation,
  $$\begin{array}{@{}rcl@{}} \Lambda_{42}(s|z_{i},w_{i},u_{i}) + \log(1-u_{42i})=0. \end{array} $$
  If *C*≤*T*_*i*_, then the *i*^th^ subject is defined as being censored at time *C* after experiencing LTF and a non-fatal event, i.e., $i \in \mathcal {B}_{5}$. Otherwise, the *i*^th^ subject is defined as being dead at time *T*_*i*_ after experiencing LTF and a non-fatal event, i.e., $i \in \mathcal {B}_{6}$.

In the simulation settings, we consider three types of regression coefficients (i.e., ‘even’, accelerated (‘acc’), and decelerated (‘dec’)) as well as three types of LTF proportions (i.e., ‘low’, ‘moderate’, and ‘high’). For the ‘even’ type, there are no differences in the effects of the covariates on the hazard rate of death before and after experiencing a non-fatal event. That is, *α*_02_=*α*_12_=0.01 and *β*_02_=*β*_12_=0.004. Meanwhile, for ‘acc’ and ‘dec’, increasing and decreasing effects are noted on the hazard rates of death, respectively. That is, *α*_02_=0.01, *α*_12_=0.0125, *β*_02_=0.004, and *β*_12_=0.005 for ‘acc’, whereas *α*_02_=0.02, *α*_12_=0.01, *β*_02_=0.008, and *β*_12_=0.004 for ‘dec’. For the rest of the regression coefficients, we set *β*_01_=*β*_03_=*β*_32_=0.004 and *α*_01_=*α*_03_=*α*_32_=0.01. Moreover, we set *θ*_03_=0.00075, *θ*_03_=0.002, and *θ*_03_=0.004 for the ‘low’, ‘moderate’, and ‘high’ types, respectively. The remaining shape parameters of the baseline transition intensities were set as *θ*_01_=*θ*_32_=0.002 and *θ*_02_=0.001. Tables 1, 2, and 3 provide the relative bias (‘r.Bias’), standard deviation (‘SD’), average of the standard errors (‘SEM’), and coverage probability (‘CP’) of 95% confidence intervals for the regression parameters and the variance estimate of the frailty distribution, respectively, according to the three LTF proportions. For comparison purposes with the proposed approach (‘proposed’), each table also displays the results obtained by simply assuming that a non-fatal event occurred at the end of the right endpoint of the interval (‘imputed-by-the-right-endpoint’). When the type of the regression coefficients is fixed at ‘even’, ‘acc’, or ‘dec’, the CPs of the regression parameters corresponding to the ‘proposed’ case are close to a nominal level of 0.95 irrespective of the LTF proportions, whereas those of the regression parameters such as *b*_01_ and *b*_03_, are much smaller than 0.95 for the ‘imputed-by-the-right-endpoint’ case. For the results based on the ‘proposed’ method, as the proportion of LTF increases, the mean squared error (MSE) for estimates of some regression parameters (e.g., *a*_03_,*a*_32_, and *b*_32_) decreases, while the MSE of other regression parameters (e.g., *a*_02_,*b*_02_, and *b*_03_) increases, regardless of the type of regression coefficients.

**Table 1:** Empirical results for the averages of the relative bias (r.Bias) and standard errors (SEM), standard deviation (SD), and coverage probability (CP) for the regression parameters and variance parameter of the log-normal frailty based on the ‘imputed-by-the-right-endpoint’ and ‘proposed’ methods when the type of regression coefficients is ‘even’ under three types of LTF proportions (‘low’, ‘moderate’, and ‘high’)

		Low (LTF(%)=22.6)				Moderate (LTF(%)=34.2)				High (LTF(%)=47.3)			
Parameter	True value	r.Bias (%)	SD (×10^5^)	SEM (×10^5^)	CP (%)	r.Bias (%)	SD (×10^5^)	SEM (×10^5^)	CP (%)	r.Bias (%)	SD (×10^5^)	SEM (×10^5^)	CP (%)
		*Imputed-by-the-right-endpoint method*											
*α*_01_	0.01	81.7	15026	15104	96.8	73.3	15105	14975	95.2	20.6	14450	15186	96.2
*α*_02_	0.01	176.2	22297	22961	95.4	324.5	24408	24066	94.8	28.8	27643	27426	95.4
*α*_03_	0.01	133.2	27006	26429	95.4	86.1	16223	17086	96.2	75.3	13291	13250	95.2
*α*_12_	0.01	-145.7	28481	25849	95.2	-113.4	29996	26279	93.6	74.1	27309	25620	96.2
*α*_32_	0.01	109.7	65489	55615	93.2	77.1	36087	33747	96.8	50.8	26680	25219	94.8
*β*_01_	0.004	-10.1	81	87	93.8	-9.4	80	88	91.6	-8.6	87	88	92.6
*β*_02_	0.004	1.1	92	92	94.6	-1.4	97	96	94.4	-0.4	103	101	94.0
*β*_03_	0.004	-7.2	87	86	91.2	-6.4	105	105	93.4	-9.3	136	133	91.6
*β*_12_	0.004	3.5	115	115	97.0	5.7	112	116	95.6	3.4	116	115	95.8
*β*_32_	0.004	-0.3	217	199	96.8	7.6	174	173	96.4	5.3	148	152	97.2
*σ* ^2^	0.01	904.5	8164	8782	93.8	815.5	7848	8084	92.4	771.6	7626	7076	90.4
		*Proposed method*											
*α*_01_	0.01	32.2	15236	15136	96.2	64.8	15344	15084	95.0	20.7	14496	15279	96.2
*α*_02_	0.01	70.2	22692	22951	95.0	286.1	24010	24133	95.2	100.4	28030	27569	95.2
*α*_03_	0.01	289.4	27525	26349	95.0	121.3	16740	17161	95.0	86.3	13465	13391	95.0
*α*_12_	0.01	-24.6	26718	25661	95.8	-83.4	29355	26230	94.0	-37.0	27102	25736	96.2
*α*_32_	0.01	123.2	67188	56122	93.6	93.4	36291	33577	95.6	54.0	26591	25204	94.2
*β*_01_	0.004	-5.5	89	90	95.0	-5.0	85	91	93.8	-5.3	88	91	94.4
*β*_02_	0.004	5.9	95	97	96.2	4.0	99	100	94.2	4.8	107	106	94.8
*β*_03_	0.004	-1.7	92	91	93.2	-1.7	107	110	95.6	-3.3	141	139	93.4
*β*_12_	0.004	-1.9	109	110	96.4	0.9	108	112	95.2	0.1	110	111	96.0
*β*_32_	0.004	1.0	214	199	96.6	8.4	179	175	96.2	4.4	150	153	97.0
*σ* ^2^	0.01	1016.2	9069	9074	92.2	897.8	7743	8198	91.0	874.1	7616	7423	87.2

**Table 2:** Empirical results for the averages of the relative bias (r.Bias) and standard errors (SEM), standard deviation (SD), and coverage probability (CP) for the regression parameters and variance parameter of the log-normal frailty based on the ‘imputed-by-the-right-endpoint’ and ‘proposed’ methods when the type of regression coefficients is ‘dec’ under three types of LTF proportions (‘low’, ‘moderate’, and ‘high’)

		Low (LTF(%)=19.4)				Moderate (LTF(%)=31.4)				High (LTF(%)=43.5)			
Parameter	True value	r.Bias (%)	SD (×10^5^)	SEM (×10^5^)	CP (%)	r.Bias (%)	SD (×10^5^)	SEM (×10^5^)	CP (%)	r.Bias (%)	SD (×10^5^)	SEM (×10^5^)	CP (%)
		*Imputed-by-the-right-endpoint method*											
*α*_01_	0.01	36.6	15548	14973	93.8	-110.5	15890	15137	94.2	-97.0	15327	15425	94.6
*α*_02_	0.02	-8.8	23990	22697	96.0	2.5	23708	25122	97.0	44.4	29732	28452	95.2
*α*_03_	0.01	400.1	27926	26768	95.8	9.1	18791	17136	93.8	-80.3	14385	13500	93.6
*α*_12_	0.01	100.0	27898	26371	94.6	199.2	28271	25833	92.8	172.0	29132	26387	93.8
*α*_32_	0.01	274.6	66261	56538	93.6	121.0	35562	33829	94.4	125.5	29879	26193	94.2
*β*_01_	0.004	-6.6	99	97	90.0	-8.7	89	96	93.4	-6.3	95	97	95.2
*β*_02_	0.008	-0.9	137	139	94.0	-2.8	129	140	95.8	-0.3	143	149	96.0
*β*_03_	0.004	-2.8	96	98	93.0	-5.7	121	116	91.0	-9.5	142	144	94.4
*β*_12_	0.004	6.4	120	125	97.2	6.2	120	127	98.4	5.8	124	125	96.8
*β*_32_	0.004	0.6	234	218	96.8	7.6	204	188	96.4	9.5	177	169	95.0
*σ* ^2^	0.01	631.4	7287	8545	97.6	705.2	6851	7982	96.2	714.8	7217	7540	90.4
		*Proposed method*											
*α*_01_	0.01	21.6	15586	15134	94.0	-56.8	16268	15285	94.0	-92.4	15530	15487	94.2
*α*_02_	0.02	-0.6	24316	22833	95.8	-18.9	24116	25162	96.6	104.0	30008	28458	95.0
*α*_03_	0.01	337.6	27943	26924	95.4	-9.4	18629	17265	94.0	-83.6	14279	13600	94.2
*α*_12_	0.01	43.2	27653	26452	94.6	175.4	27650	25973	93.8	102.5	28481	26251	93.2
*α*_32_	0.01	296.4	67196	57028	93.4	140.3	36513	34261	94.8	170.0	30037	26145	93.8
*β*_01_	0.004	-2.5	106	101	91.8	-4.5	95	99	94.6	-2.3	100	100	95.8
*β*_02_	0.008	3.8	146	146	95.6	2.0	133	148	98.6	3.6	148	155	97.0
*β*_03_	0.004	1.3	102	102	93.4	-0.3	124	121	94.0	-3.0	149	150	95.4
*β*_12_	0.004	0.3	112	119	96.0	0.8	116	122	98.0	1.1	117	121	96.4
*β*_32_	0.004	2.5	232	222	97.2	8.5	204	189	96.4	9.7	178	170	95.6
*σ* ^2^	0.01	838.4	8153	9217	94.2	852.3	7505	8446	93.8	791.2	7447	7745	89.2

**Table 3:** Empirical results for the averages of the relative bias (r.Bias) and standard errors (SEM), standard deviation (SD), and coverage probability (CP) for the regression parameters and variance parameter of the log-normal frailty based on the ‘imputed-by-the-right-endpoint’ and ‘proposed’ methods when the type of regression coefficients is ‘acc’ under three types of LTF proportions (‘low’, ‘moderate’, and ‘high’)

		Low (LTF(%)=22.3)				Moderate (LTF(%)=34.2)				High (LTF(%)=47.6)			
Parameter	True value	r.Bias (%)	SD (×10^5^)	SEM (×10^5^)	CP (%)	r.Bias (%)	SD (×10^5^)	SEM (×10^5^)	CP (%)	r.Bias (%)	SD (×10^5^)	SEM (×10^5^)	CP (%)
		*Imputed-by-the-right-endpoint method*											
*α*_01_	0.01	3.3	14848	14915	95.2	-20.8	16313	15029	92.0	-63.6	15651	15144	95.0
*α*_02_	0.01	140.0	22910	22360	94.0	23.4	24852	24383	95.0	9.0	26169	28020	97.2
*α*_03_	0.01	79.0	25396	26622	96.2	115.9	18132	17194	92.8	-4.9	14262	13148	93.0
*α*_12_	0.0125	-100.2	27253	26397	94.4	10.5	30146	26715	93.0	74.9	25881	25844	95.4
*α*_32_	0.01	479.4	59836	50929	92.0	-108.0	37118	33609	95.2	125.6	26069	25027	94.6
*β*_01_	0.004	-11.6	89	86	89.4	-10.8	92	87	88.6	-11.3	86	87	89.2
*β*_02_	0.004	-1.2	83	92	96.4	-3.9	89	94	95.2	-0.5	102	101	92.8
*β*_03_	0.004	-8.6	83	85	91.4	-10.3	109	103	90.8	-11.6	149	133	89.4
*β*_12_	0.005	3.7	133	130	96.0	2.3	125	126	96.2	3.0	131	129	96.0
*β*_32_	0.004	0.4	197	199	95.8	6.6	172	171	95.2	3.4	139	151	96.2
*σ* ^2^	0.01	878.9	8926	8822	92.6	734.8	6989	7754	91.4	775.5	7514	7231	87.6
		*Proposed method*											
*α*_01_	0.01	29.1	15161	15024	95.2	11.9	16449	15111	91.8	-99.5	15704	15169	94.8
*α*_02_	0.01	126.2	22920	22435	93.8	-49.7	25179	24488	95.2	-34.2	26495	28218	97.4
*α*_03_	0.01	9.4	26768	26779	95.8	98.7	17948	17287	93.8	1.1	14013	13191	93.8
*α*_12_	0.0125	21.2	26832	26404	95.2	44.7	29934	26567	92.8	52.4	25781	25861	95.6
*α*_32_	0.01	280.8	60729	51753	91.4	-181.5	36859	33796	95.4	173.3	26819	25056	93.2
*β*_01_	0.004	-7.5	95	90	92.2	-6.8	95	90	90.8	-8.1	89	90	93.0
*β*_02_	0.004	4.1	88	96	97.2	1.8	94	99	96.2	4.6	106	105	93.4
*β*_03_	0.004	-3.9	88	89	93.6	-4.1	116	108	92.8	-6.3	154	138	90.6
*β*_12_	0.005	-0.9	124	124	96.4	-2.0	117	122	97.0	-1.2	123	124	95.4
*β*_32_	0.004	2.3	195	200	95.8	6.9	171	172	95.6	3.8	137	152	97.2
*σ* ^2^	0.01	999.2	9282	9124	93.0	936.4	8781	8219	88.6	873.0	8002	7404	85.6

Sensitivity analysis is also conducted to investigate how the estimator of the parameter behaves with different frailty distributions. For simplicity of computation, we consider only the ‘even’ case for the regression parameters and the ‘moderate’ LTF proportion. Three different frailty distributions are used, along with a normal distribution with a mean of zero and a variance of 0.01. These are uniform, double exponential, and gamma distributions with specific parameter value(s), for which the mean and variance of each distribution are the same as those of the normal distribution. Simulation results are provided in Table 4. We compare the results of the three distributions with those of the normal distribution. The uniform and double exponential distributions are symmetric, like the normal distribution. However, the uniform distribution has thinner tails than the normal distribution, while the double exponential distribution has heavier tails than the normal distribution. Alternatively, unlike the normal distribution, the gamma distribution is an asymmetric distribution. Overall, there are no differences in the values of r.Bias and CP between the three distributions and the normal distribution. This implies that the proposed estimators are robust to misspecifications of the frailty distribution.

**Table 4:** Sensitivity analysis of the ‘proposed’ method depending on the underlying frailty distribution in terms of the averages of the relative bias (r.Bias) as well as the standard errors (SEM) and coverage probability (CP) when the type of the regression coefficients is ‘even’ and the LTF proportion is ‘moderate’

		*N*(0,0.01)			*U*(−0.173,0.173)			*DE*(0.007)			*G*(100.5,0.01)		
		(LTF(%)=34.2)			(LTF(%)=34.4)			(LTF(%)=34.5)			(LTF(%)=34.5)		
Parameter	True value	r.Bias (%)	SEM (×10^5^)	CP (%)	r.Bias (%)	SEM (×10^5^)	CP (%)	r.Bias (%)	SEM (×10^5^)	CP (%)	r.Bias (%)	SEM (×10^5^)	CP (%)
*α*_01_	0.01	18.8	15299	94.0	40.9	15169	94.2	36.7	15215	94.8	-28.7	15089	96.2
*α*_02_	0.01	1.3	24633	93.2	-83.8	24639	97.0	89.0	24401	94.4	45.0	24623	96.6
*α*_03_	0.01	-143.8	17210	94.4	58.9	17266	93.8	-110.5	16925	93.8	119.9	17198	93.6
*α*_12_	0.01	181.1	25925	96.4	-27.9	26141	94.8	27.1	26318	93.2	159.3	25682	95.2
*α*_32_	0.01	-16.5	33404	95.4	-80.1	33158	94.0	-22.5	33619	94.0	-82.4	33595	91.8
*β*_01_	0.004	-4.3	91	94.8	-5.8	90	94.0	-3.3	91	94.0	-6.7	90	92.2
*β*_02_	0.004	2.7	99	96.4	4.9	100	94.8	5.4	101	97.0	4.4	99	95.4
*β*_03_	0.004	-2.2	109	93.2	-2.9	109	91.0	-2.8	110	94.4	-2.9	108	91.8
*β*_12_	0.004	0.1	111	96.6	2.3	111	95.0	0.6	110	94.8	-0.3	111	95.6
*β*_32_	0.004	8.0	174	97.0	0.8	169	96.0	2.9	171	96.8	5.2	170	96.4
*σ* ^2^	0.01	955.8	8431	89.2	974.2	8325	89.4	926.1	8284	89.6	928.7	8462	89.2

### Illustrative data analysis

PAQUID data were collected to investigate the effects of dementia on mortality. Samples were taken from community residents of two southwestern regions (Gironde and Dordogne) of France [7]. The population consists of elderly people of ages 65 or above, between 1988 and 1990, whose socio-demographic characteristics and mental health status were recorded every two to three years. A total of 3675 persons was selected to participate in the study; among these individuals, 832 (22.6%) were diagnosed with dementia, 639 of whom died. The remaining 2843 participants (77.4%) did not experience dementia but 2298 of them died.

In this article, we performed an analysis based on ‘paq1000’ data, which included 1000 randomly selected observations from the PAQUID data [21]. The paq1000 data consist of several pieces of information, such as the mental health status (diagnosed with dementia or dementia-free), dead or alive status, age (including a n’s age at the start of study, their age at the last dementia-free visit, their age when they were diagnosed with dementia, their age at their time of death, and their age at censoring), gender, and educational background (educated or non-educated in terms of graduation of elementary school, say certificate). When a person who was not diagnosed with dementia at their last visit has not been traced for more than four years, this person is assigned to the LTF category. Table 5 shows summary statistics to briefly grasp the subjects’ characteristics including ages at entry, at dementia diagnosis, at death after dementia, at death without dementia, and at death after LTF. A total of 231 persons was categorized as LTF; among these, 159 (68.8%) died. Moreover, 127 (68.3%) persons out of the 186 who experienced dementia died, and 438 (75.1%) persons out of the 583 dementia-free persons died. Moreover, age at dementia diagnosis is higher for women than for men regardless of the education group (with certificate or without certificate). The same trend is observed both at age after dementia and at age without dementia. Meanwhile, age at death after LTF is a bit higher for women than for men.

**Table 5:** Patients’ characteristics of ages at entry, at demensia (DM) diagnosis, at death after DM, at death without DM, and at death after LTF

			Gender							
			Women				Men			
			Certificate				Certificate			
	All		With		Without		With		Without	
		mean		mean		mean		mean	*n*	mean
Age	*n*	(±SD)	*n*	(±SD)	*n*	(±SD)	*n*	(±SD)	*n*	(±SD)
at entry	1000	75.0	456	76.0	122	74.4	306	74.7	116	72.8
		(±6.84)		(±7.03)		(±7.02)		(±6.67)		(±5.61)
at DM diagnosis	186	83.3	109	84.2	17	83.3	45	81.5	15	81.9
		(±5.46)		(±5.60)		(±5.39)		(±4.92)		(±4.99)
at death after DM	127	87.6	72	88.8	10	88.9	36	85.4	9	84.8
		(±5.93)		(±6.21)		(±5.19)		(±5.23)		(±4.12)
at death without DM	438	84	170	85.9	47	84.5	161	82.8	60	81.1
		(±7.03)		(±7.17)		(±6.91)		(±6.44)		(±6.78)
at death after LTF	159	87.2	80	87.7	20	86.3	43	87.3	16	85.9
		(±6.42)		(±6.04)		(±6.85)		(±6.75)		(±7.12)

**Fig. 2:**
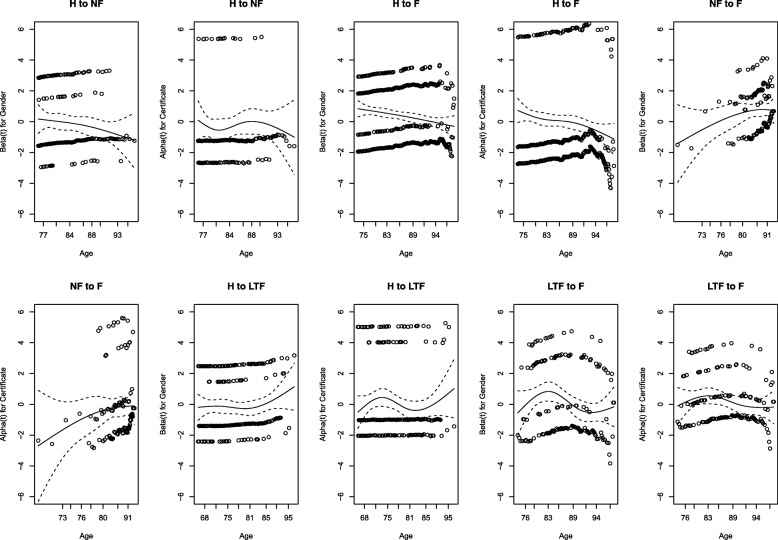
Diagnostic plots showing the constancy of the coefficients in the PAQUID data. Each plot shows a component of the time-varying coefficient against the ordered time. A spline smoother (solid line) is shown together with the ±2 standard deviation bands (dashed lines)

First, we check to see whether each covariate satisfies the proportional hazards assumption by using the test procedure of [22] and the Schoenfeld residual [23]. Figure 2 shows diagnostic (scattered) plots of the scaled Schoenfeld residual versus age. In each plot, we mark a spline smoother (solid line) as well as two standard deviation bands (dashed lines). In the curve showing the effect of gender, there is a decreasing trend on transitions 0→1 and 0→2, an increasing trend on 1→2 and 3→2, and a quite steady pattern for 0→3. In the curve showing the effect of certificate, there is a prominent decreasing trend for transition 0→2, while the other transitions show steady patterns. Table 6 provides the *p*-values for the test results [11]. For the gender effect, only the *p*-value for transition 0→3 is greater than 0.1, which seems to violate the proportional hazards assumption. Alternatively, the *p*-values for all transitions for the certificate effect, with the exception of 0→2, are greater than 0.1. This seems to satisfy the proportional hazards assumption. Thus, we put ‘gender’ into the additive side and ‘certificate’ into the multiplicative side for future analyses:

$$\begin{array}{@{}rcl@{}} \lambda_{rs}(t|z&=&\text{gender},w=\text{certificate},u)\\&=&\exp(u)\left\{\beta_{rs}z+\exp(\alpha_{rs}w)\theta_{rs}\gamma_{rs}t^{\gamma_{rs}-1}\right\} \end{array} $$

**Table 6:** *P*-values of the test used to check the proportional hazard assumption for each transition model

Covariate	Transition models				
	0→1	0→2	1→2	0→3	3→2
Gender	0.063	<0.001	0.093	0.354	0.062
Certificate	0.963	<0.001	0.147	0.754	0.148

for $(r,s)\in \mathcal {A}.$

**Fig. 3:**
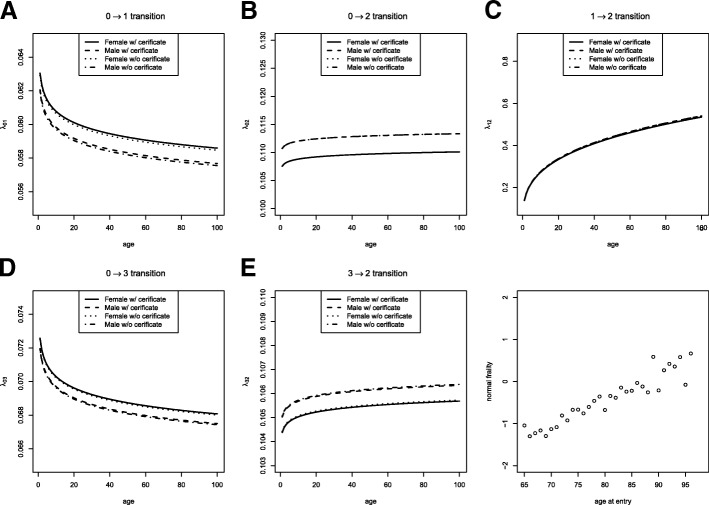
Five transition intensities over age by gender and certificate: 0→1, 0→2, 1→2, 0→3, 3→2 transitions and estimated normal frailties of each cluster classified by age at entry

Table 7 shows a summary of the estimation procedures. We provide the estimates of the regression coefficients along with their standard errors and *p*-values. For the transition from H to LTF (0→3), the intensities for women are larger than for men, with a value of 0.00849 (849 out of 100000 persons). However, this turned out to be insignificant with a *p*-value of 0.439. For the intensity of the 3→2 transition, a reversed outcome was obtained, with a value of 0.00619 for men with a very significant *p*-value of less than 0.001. For the 0→1 transition, women showed a larger intensity than men with a value of 0.0156, yielding a non-significant result with a *p*-value of 0.245. For the 1→2 transition, the intensity for men is similar to that for women, with a value of 0.0101 and a *p*-value of 0.961. Finally, for the 0→2 transition, the intensity for men is larger than for women with a value of 0.0295 along with a significant *p*-value of 0.038. Meanwhile, all transition intensities of the non-educated group are similar to those of the educated group. Finally, the estimate for the variance *σ*^2^ on the common frailty is 0.999 with a highly significant *p*-value less than 0.001, showing non-homogeneity between clusters classified by the age at entry. Figure 3 shows five transition intensities over age by gender and certificate and estimated normal frailties of each cluster. As presented in Fig. 3, the transition intensities of 0→1 and 0→3 are higher for women than for men over age regardless of the education group, while these trends are reversed for the 0→2 and 3→2 transitions. For the transition 1→2, no difference is observed between women and men, but there is a significantly monotone increasing trend over age. These are consistent with the results in Table 7.

**Table 7:** Regression parameter estimates (Est) with the accompanying standard errors (SE) and *p*-values (*P*)

Covariate	Param	Est	SE	*P*
Gender	*β*_01_	-0.0156	0.0132	0.245
	*β*_02_	0.0295	0.0136	0.004
	*β*_12_	0.0101	0.205	0.961
	*β*_03_	-8.49 ×10^−3^	0.0108	0.439
	*β*_32_	6.19 ×10^−3^	1.57 ×10^−3^	<0.001
Certificate	*α*_01_	-2.10 ×10^−3^	0.190	0.991
	*α*_02_	-3.00 ×10^−5^	0.128	0.999
	*α*_12_	3.90 ×10^−5^	0.621	0.999
	*α*_03_	-9.80 ×10^−4^	0.151	0.995
	*α*_32_	3.85 ×10^−4^	1.133	0.999
	*σ* ^2^	0.999	2.55 ×10^−3^	<0.001

## Conclusions

We considered a multi-state model for semi-competing risks data, for which there exists information on fatal events, but information on non-fatal events may not be available due to lost to follow-up. More precisely, we proposed an additive and multiplicative random effect model by combining the additive risk model [16] with the proportional hazards model [8, 9] in order to derive the conditional likelihood function. An adjusted importance sampling method was used to compute the marginal likelihood function, where the MLEs for the regression coefficients were obtained by an iterative quasi-Newton algorithm. The proposed model was illustrated using PAQUID data and yielded several promising results. The risk of transition from a healthy state to dementia is higher for women. However, the risk of death is higher for men regardless whether a subject is diagnosed with dementia or not. Meanwhile, the risk of transition from a healthy state to dementia is higher for the educated group. The risk of death after being diagnosed with dementia is higher for the educated group; however, a reversed result is observed for non-diagnosed subjects. Furthermore, we conducted simulations with finite-sample sizes to investigate the efficiency of the proposed estimators. In particular, we considered nine combinations of three different types of regression coefficients and three different types of LTF proportions. In general, the coverage probabilities of the regression parameters are close to a nominal level of 0.95 in most cases. Moreover, according to a referee’s suggestion, we investigated influence of parameter estimation when LTF is omitted (not censoring) in our simulation studies. Finally, we performed simulations with the same realizations generated from each configuration included in Tables 1, 2, and 3. Based on the results not reported here, the CP of parameter *β*_01_ is extremely lower than a nominal level of 0.95 for all configurations. In addition, when the type of regression coefficients is ‘dec’, the CP of parameter *β*_02_ is much smaller than 0.95 regardless of types of LTF proportions. Moreover, compared to each table, namely, Tables 1, 2, and 3, the relative bias of *β*_01_ increases around ten times for all configurations. This is the reason omitting LTF results in route changes of subjects included in routes 5 and 6 at the data generation stage, i.e., from route 5 to 1 (when the fatal event is censored) or from route 6 to 2 (when the fatal event is observed) according to the fatal status of subjects.

The proposed model has some drawbacks. At the initial stage of this research, as developed in [8, 9, 16], we intended to consider a semi-parametric model incorporating five transition intensity models. However, it was quite difficult to handle nonparametric estimation procedures for the baseline transition intensity associated with the nuisance parameter because the total number of parameters is proportional to the number of subjects. Rather, we assumed a Weibull distribution for the baseline transition intensity; further research should be carried out to avoid the use of this specific distribution. To circumvent arbitrariness, it is necessary to calculate the Nelson-Aalen estimators for the cumulative baseline transition intensity [8, 9, 16] and extend this method to semi-competing risks models based on the profile likelihood function. Another plausible remedy would be to apply a spline smoothing method on the baseline transition intensity proposed by [24, 25]. In the additive and multiplicative hazards model with frailty, one could use a semi-parametric Bayesian approach by assuming a prior distribution on the log-normal frailty. Subsequently, conventional Markov Chain Monte Carlo computation would be proceeded for the full conditional distribution on the frailty [26, 27].

## Abbreviations

CP: Coverage probability
F: Fatal
H: Healthy
LTF: Lost-to-follow-up
MSE: Mean squared error
NF: Non-fatal
PAQUID: Personnes Agées Quid
r.Bias: Relative bias
SD: Standard deviation
SEM: Average of the standard errors
